# Phase I dose-escalation study of the safety, tolerability, and pharmacokinetics of aflibercept in combination with S-1 in Japanese patients with advanced solid malignancies

**DOI:** 10.1007/s10637-019-00888-z

**Published:** 2020-01-06

**Authors:** Toshihiko Doi, Narikazu Boku, Yusuke Onozawa, Keishiro Takahashi, Osamu Kawaguchi, Atsushi Ohtsu

**Affiliations:** 1 Department of Gastrointestinal Oncology, National Cancer Center Hospital East, 6-5-1, Kashiwanoha, Kashiwa, Chiba, 277-8577 Japan; 2 Department of Gastrointestinal Medical Oncology, National Cancer Center Hospital, Tokyo, Japan; 3 Division of Clinical Oncology Shizuoka Cancer Center, Shizuoka, Japan; 4 Research & Development, Sanofi K.K., Tokyo, Japan; 5 Biostatistics & Programming, Sanofi K.K., Tokyo, Japan

**Keywords:** Aflibercept, S-1, Phase I trial, Japanese, VEGF trap

## Abstract

*Background* Aflibercept, a recombinant fusion protein binding VEGF-A, VEGF-B and placental growth factor, inhibits tumor growth by blocking angiogenesis. The aim of this phase I dose-escalation study was to determine the recommended phase II dose (RP2D) of aflibercept in combination with S-1 in Japanese patients with solid tumors. *Patients and methods* Sequential cohorts of 3–6 patients with metastatic or unresectable solid tumors, who had failed at least one prior line of standard treatment or who were not suitable for such treatment, were to receive escalating doses of aflibercept every 2 weeks, starting at 2 mg/kg, combined with S-1 at 40 mg/m^2^ twice daily (80 mg/m^2^/day; 4 weeks on/2 weeks off). Dose-escalation was to be based on the incidence of dose-limiting toxicity (DLT). Blood samples were collected for pharmacokinetic analysis. *Results* At the first dose level (aflibercept 2 mg/kg plus S-1) 1 of 6 patients experienced a DLT (grade 4 proteinuria). The aflibercept dose was consequently escalated to 4 mg/kg; 1 of 3 patients treated at this dose level had a DLT (grade 2 pleural effusion), and another patient experienced grade 3 reversible posterior leukoencephalopathy syndrome after the DLT assessment period. Additional patients were therefore enrolled into the first dose level to explore safety and tolerability. The study was subsequently terminated prematurely. The maximum tolerated dose was not reached and the RP2D was not determined in Japanese patients. *Conclusions* The tolerability and safety of aflibercept 2 mg/kg in combination with S-1 was confirmed in Japanese patients with advanced solid tumors.

## Introduction

The process of angiogenesis plays a crucial role in tumor growth and metastasis [1]. New blood vessels from existing vasculature maintain a source of nutrition and oxygen for the tumor from the host. Although the mechanism of angiogenesis is complex, involving multiple signaling pathways, the proangiogenic cytokine, vascular endothelial growth factor A (VEGF-A), is of key importance [2]. VEGF-A is a homodimeric protein which binds to and activates two high-affinity receptors, VEGFR-1 (also known as FLT-1) and VEGFR-2 (also known as KDR) [3]. Furthermore, VEGF-A acts as a powerful mitogen for endothelial cells and increases vessel permeability very potently. Thus, VEGF-A promotes the formation of new vessels that are required for tissue growth [2, 3].

VEGF-A has been reported to be overexpressed in several types of human cancer and associated with increased tumor vascularity, proliferation, progression, invasion, metastasis, and poor prognosis [4, 5]. Therefore, VEGF-A is a major target for anti-angiogenic therapy. Clinical studies with bevacizumab, an anti-VEGF-A antibody, have shown that targeting this growth factor is effective for the clinical management of metastatic colorectal cancer (mCRC) [6, 7], advanced non-small cell lung cancer [8], metastatic renal cancer [9, 10], and glioblastoma multiforme [11].

Aflibercept (also known as ziv-aflibercept in the United States; aflibercept beta in Japan) is a soluble, decoy receptor construct which incorporates the second immunoglobulin (Ig)-like domain of VEGFR-1 joined to the third Ig-like domain of VEGFR-2, which are fused to the Fc portion of human IgG1 [12, 13]. This construction allows aflibercept to bind all isoforms of VEGF-A at subpicomolar affinity levels [14]. In addition, aflibercept also binds two more growth factors, placenta growth factor (PlGF) and VEGF-B [15]. Data from patient-derived colorectal cancer xenograft models have shown aflibercept to exhibit greater antitumor activity than bevacizumab [16]. The VELOUR study, a large randomized, placebo-controlled, phase III trial, demonstrated that addition of aflibercept to infusional 5-fluorouracil (5-FU), folinic acid and irinotecan (FOLFIRI) significantly improved overall survival compared with placebo plus FOLFIRI in patients with mCRC previously treated with an oxaliplatin-containing regimen [17]. Aflibercept was administered intravenously in this study, which enrolled predominantly Caucasian patients, at a dose level of 4 mg/kg every 2 weeks. A phase I study has demonstrated that FOLFIRI plus aflibercept at a dose level of 4 mg/kg every 2 weeks has a manageable toxicity profile, pharmacokinetic (PK) parameters consistent with findings in Caucasian patients, and promising efficacy in Japanese patients with mCRC previously treated with at least one chemotherapy regimen [18].

Preclinical assessment of aflibercept with 5-FU in tumor-bearing mice showed that the combination was synergistic and that there was no overlap in host toxicity (Sanofi, data on file). S-1 is an oral fluoropyrimidine formulation including tegafur, a prodrug of 5-FU; gimeracil (5-chloro-2,4-dihydroxypyridine [CDHP]), an inhibitor of 5-FU catabolism; and oteracil, which decreases 5-FU activation in the gut, thereby reducing gastrointestinal toxicity.

The primary objective of this phase I dose-escalation study was to determine the recommended phase II dose (RP2D) of aflibercept that could be safely administered intravenously once every 2 weeks in combination with S-1 in Japanese patients.

## Materials and methods

### Study population

Patients aged ≥20 years, with a histologically or cytologically confirmed solid malignancy that was recurrent or unresectable, for which S-1 treatment had regulatory approval in Japan, were eligible for inclusion. They must also have had failure of at least one prior line of standard treatment or be unsuitable for standard care, an Eastern Cooperative Oncology Group (ECOG) performance status ≤2; adequate organ function (hemoglobin ≥9.0 g/dL; absolute neutrophil count ≥1.5 × 10^9^/L; platelets ≥100 × 10^9^/L; creatinine ≤1.0 x upper limit of normal (ULN) or creatinine clearance calculated according to the Cockroft-Gault formula ≥60 mL/min if between 1.0 to ≤1.5 x ULN; either proteinuria ≤500 mg/24 h or urine protein:creatinine ratio ≤1; aspartate aminotransferase and alanine aminotransferase ≤2.5 x ULN, total bilirubin ≤1.5 x ULN, and serum albumin ≥3.0 g/dL). All toxic effects of prior anticancer therapy, excluding alopecia, must have resolved to grade ≤1.

Exclusion criteria were: a diagnosis of squamous cell lung cancer; an anticipated need for a major surgical procedure or radiation therapy during the study; history of hypersensitivity to recombinant proteins, or fluoropyrimidines, including S-1 or severe drug allergy; treatment with chemotherapy, hormonal therapy, radiotherapy, surgery, blood products, or any investigational agent within the 28 days (42 days for nitrosourea agents, mitomycin C, or immunotherapy) prior to study enrollment; known dihydropyrimidine dehydrogenase deficiency; cumulative radiation therapy to >25% of the total bone marrow; uncontrolled hypertension defined as blood pressure (BP) >150/100 mmHg on at least two repeat determinations on separate days within 4 weeks prior to enrollment; severe cardiac, cerebral or gastrointestinal or thromboembolic events within 180 days prior to study entry; history of brain metastases, spinal cord compression, or carcinomatous meningitis, or new evidence of brain or leptomeningeal disease on screening computed tomography (CT) or magnetic resonance imaging (MRI) scan; peritoneal metastases clearly detectable by CT or MRI; malignant ascites requiring drainage; active infection, hepatitis C virus, hepatitis B virus surface antigen positive or on antiviral therapy for human immunodeficiency virus; clinically significant bleeding diathesis or underlying coagulopathy; administration of warfarin; pregnant or breast-feeding; prior treatment with an investigational product (prior treatment with S-1 was permitted, unless inclusion was inappropriate for safety reasons).

### Study design

This was a dual-center, open-label, sequential-cohort dose-escalation study of aflibercept administered intravenously every 2 weeks in combination with S-1 in Japanese patients with advanced solid malignancies. The study was designed as a hybrid phase I study starting with single agent therapy proceeding to combination therapy, comprising an aflibercept single-agent 2-week run-in phase followed by a combination phase with S-1. The primary objective of the study was to determine the RP2D of aflibercept in combination with S-1 in Japanese patients. Secondary objectives included assessments of safety, dose-limiting toxicity (DLT), PK, antitumor activity and the immunogenicity of aflibercept.

The protocol was approved by the Institutional Review Boards at both participating centers and the study was conducted in accordance with the ethical principles laid out in the Declaration of Helsinki. All patients provided written informed consent prior to enrollment.

### Drug dose and administration

The planned starting dose of aflibercept was 2 mg/kg every 2 weeks, based on previous studies in other populations. Two further dose levels were planned: 4 mg/kg every 2 weeks and 6 mg/kg every 2 weeks. Aflibercept was to be administered intravenously over 1 h according to the assigned dose level every 2 weeks. Cycle 1 extended over 8 weeks, with subsequent cycles being 6 weeks. In the first 2 weeks of cycle 1 (single-agent phase), patients initially received a single administration of aflibercept , and then from day 15, patients received aflibercept in combination with oral S-1 at 40 mg/m^2^ twice daily (80 mg/m^2^/day), administered to day 42, followed by 2 weeks off. In subsequent 6-week cycles, S-1 was administered on days 1 to 28 (4 weeks on/2 weeks off) with aflibercept administered every 2 weeks. From the starting dose level of 2 mg/kg, sequential cohorts of 3–6 patients were to be treated at escalating dose levels of aflibercept, while the dose of S-1 was constant at 40 mg/m^2^ twice daily. The study followed a standard 3 + 3 design and dose escalation was to be based on the occurrence of DLT (<33% of all evaluable patients at a particular dose level) during the first cycle. Intrapatient dose escalation was not permitted.

The maximum tolerated dose (MTD) was defined as the lowest dose level at which ≥33% of all evaluable patients experienced DLT in cycle 1. If the MTD was reached, then the highest dose level below the MTD would be the RP2D. The RP2D of aflibercept in combination with S-1 was to be therefore the highest aflibercept dose at which 0 of 3 or 1 of 6 (<33%) of all evaluable patients experienced DLT during the first cycle. To further explore the safety and preliminary efficacy profile of the RP2D, the cohort receiving RP2D was to be expanded by up to 10 additional patients. If the MTD was not reached, the highest dose level cohort was to be expanded by 7 additional patients to further explore the safety of the combination; the RP2D would subsequently be determined based on safety and PK data for all patients at that dose level.

### Safety assessments and definition of DLT

Adverse events were graded according to the National Cancer Institute Common Terminology Criteria for Adverse Events version 3.0. Treatment-emergent adverse events (TEAEs) were defined as adverse events that were reported by the site investigator during the on-treatment period (from the start of treatment up to 30 days after the last dose of aflibercept). DLT was defined as any of the following events observed during the first treatment cycle: grade 3 or 4 neutropenia complicated by fever (≥38.5 °C) or infection; grade 4 neutropenia persisting for at least 7 days; grade 4 thrombocytopenia, or grade 3 thrombocytopenia complicated by hemorrhage; any grade 3 non-hematological adverse event except fatigue, anorexia, nausea, vomiting, hyponatremia (unless these adverse events was subsequently judged to be DLTs by the Study Committee, considering their frequency, duration, or requirement for excessive supportive therapy); any grade 4 non-hematological toxicity; uncontrolled hypertension defined as systolic BP >150 mmHg or diastolic BP >100 mmHg (or > 180/90 mmHg if the patient had a history of pre-existing systolic hypertension) despite 4 weeks of medical management; urinary protein excretion of >3.5 g per day that does not recover to <2.0 g per 24 h within 2 weeks; symptomatic arterial thromboembolic events including cerebrovascular accidents, myocardial infarctions, transient ischemic attacks, new onset or worsening of pre-existing angina.

### Efficacy assessment

Following a baseline evaluation by CT or MRI scans covering the head, chest, abdomen and pelvis, tumor response was assessed using the same method on day 15 and on day 42 of every treatment cycle beyond cycle 1. Tumor assessment was added to confirm a partial or complete response (4–6 weeks after initial documentation of response), whenever disease progression was suspected, and at the end of study treatment. Tumor response was evaluated according to Response Evaluation Criteria in Solid Tumors version 1.0 [19].

### Pharmacokinetics and immunogenicity

Blood samples (4.5 mL) for the analysis of plasma concentrations of free and VEGF-bound aflibercept were collected: before, at the end of, and 1, 3, 7 (day 1), 23, 29 (day 2), 47 (day 3) and 167 (day 8) hours after the end of the first infusion of aflibercept, and before administration on days 15, 29 and 42 during cycle 1 (predose). After cycle 2, blood samples were collected before each aflibercept infusion, and additionally 30 and 90 days after the last aflibercept administration. Free and VEGF-bound aflibercept levels in plasma were measured by a validated direct enzyme linked immunosorbant assay (ELISA). Concentrations of VEGF-bound aflibercept were expressed as free aflibercept equivalents (adjusted values) for PK analysis. The lower limit of quantification (LLOQ) was 31 ng/mL and 44 ng/mL (adjusted) for free and VEGF-bound aflibercept, respectively.

Blood samples (5 mL) for measuring the plasma concentrations of S-1 related compounds (tegafur, including the active metabolite of tegafur, 5-FU, CDHP and oteracil) were taken before the start of aflibercept infusion on day 42 of cycle 1, 1 h after the start of the aflibercept infusion, and 2, 4, 8, 24 and 48 h after S-1 administration. The third administration of aflibercept during cycle 1 was administered on day 42 instead of day 43 in order to evaluate the pharmacokinetic interaction with S-1. Tegafur concentration was measured by high performance liquid chromatography and 5-FU, CDHP and oteracil concentrations were measured by gas chromatography-negative ion chemical ionization mass spectrometry (GC-NICI-MS). The LLOQ of tegafur, 5-FU, CDHP and oteracil in plasma was 10 ng/mL, 1 ng/mL, 2 ng/mL and 1 ng/mL, respectively.

PK parameters calculated included: area under the concentration versus time curve extrapolated to infinity (AUC); AUC from time 0 to the real time t_last_ (AUC_last_); AUC from time 0 to 336 h (AUC_0–336_); AUC from time 0 to the end of the dosing period (AUC_τ_ – 336 h for aflibercept and 12 h for S-1); total body clearance (CL); total body clearance at steady state (CL_ss_); maximum drug concentration observed (C_max_); terminal half-life (t_1/2z_); first time to reach C_max_ (T_max_); and apparent volume of distribution at steady state (V_ss_). PK parameters were calculated using noncompartmental analysis on a validated PK data management system using WinNonlin Professional, Version 5.2.1 (Pharsight).

To screen for the presence of aflibercept antibodies in serum, blood samples (4.0 mL) were collected predose on day 1 of every odd-numbered cycle, upon study withdrawal and 90 days after study treatment discontinuation. Antibody levels were measured using a validated ELISA method, with an LLOQ of 52.7 IU/mL.

## Results

### Patients

Thirteen Japanese patients (7 female) were enrolled. Baseline characteristics are summarized in Table 1. The median age of the patients was 57 years, and all had an ECOG performance status of 0 or 1. The most common primary tumor sites were rectum (*n* = 6), colon (*n* = 4) and stomach (*n* = 2). The median number of lines of prior chemotherapy was 3 (range 2–5). Twelve patients were evaluable for DLT. One patient was not evaluable for DLT due to poor S-1 compliance. All 13 enrolled patients were evaluable for safety, PK and immunogenicity.

**Table 1:** Baseline patient and disease characteristics

Characteristic	Aflibercept dose level		All patients (*N* = 13)
	2 mg/kg (*N* = 10)	4 mg/kg (*N* = 3)	
Sex, n (%)			
Female	6 (60)	1 (33)	7 (54)
Male	4 (40)	2 (67)	6 (46)
Age, years			
Median	56.0	64.0	57.0
Range	36–73	34–74	34–74
Weight, kg			
Median	58.05	58.20	58.20
ECOG PS, n (%)			
0	7 (70)	2 (67)	9 (69)
1	3 (30)	1 (33)	4 (31)
Primary tumor site, n (%)			
Rectum	5 (50)	1 (33)	6 (46)
Colon	3 (30)	1 (33)	4 (31)
Breast	1 (10)	0	1 (8)
Stomach	1 (10)	1 (33)	2 (15)
Prior anticancer therapy,^a^ n (%)			
Chemotherapy	10 (100)	3 (100)	13 (100)
Fluoropyrimidine based	10 (100)	3 (100)	13 (100)
Anti-VEGF antibody	2 (20)	2 (67)	4 (31)
Surgery	8 (80)	3 (100)	11 (85)
Radiotherapy	1 (10)	0	1 (8)
Number of lines of prior chemotherapy, n (%)			
Median	3.0	3.0	3.0
Range	2–4	2–5	2–5

*ECOG PS*, Eastern Cooperative Oncology Group performance status

^a^A patient may have received more than one type of prior anticancer therapy

### Evaluation of DLT

At the initial 2.0 mg/kg dose level, 1 DLT (grade 4 proteinuria/nephrotic syndrome) was observed in 1 of the first 3 enrolled patients. Consequently, 3 more patients were enrolled at dose level 1. As no further DLTs occurred, the dose was increased to 4.0 mg/kg. Of the first 3 patients treated at this dose level, 1 patient experienced a grade 2 pleural effusion which was deemed to be a DLT by the Study Committee, considering the patient’s overall medical condition (bilateral pleural effusion accompanied by uncontrolled BP, proteinuria, and peripheral edema). In accordance with the protocol, 3 additional patients should therefore have been enrolled at the 4.0 mg/kg dose level. However, after the DLT assessment period, another patient developed grade 3 reversible posterior leukoencephalopathy syndrome (RPLS), which would have been considered a DLT if the event had occurred during the DLT assessment period. The sponsor discussed this with the Efficacy and Safety Evaluation Committee. Since this was the first study to administer aflibercept in Japanese patients, the Efficacy and Safety Evaluation Committee recommended that full consideration should be given to DLT events even those occurring outside the DLT evaluation period. Responding to this recommendation, the sponsor amended the protocol to allow reassessment of tolerability and safety at the 2.0 mg/kg dose level before more patients were enrolled at the 4.0 mg/kg dose level. Consequently, 4 additional patients were enrolled at the 2.0 mg/kg dose level. No further DLTs were observed. At this point, the sponsor decided to terminate the study in consideration of the global development status of S-1 treatment for gastric cancer and the progress of this study.

### Safety and tolerability

All 13 treated patients experienced at least 1 TEAE; these were predominantly grade 1 or 2. Incidences for the most common TEAEs are summarized in Table 2. At the 4 mg/kg dose level, the most common grade 3/4 TEAE was hypertension, which occurred in 2 patients. One patient in each dose level permanently discontinued study treatment due to a TEAE (grade 4 proteinuria at the 2 mg/kg dose level and grade 2 pleural effusion at the 4 mg/kg dose level). Serious TEAEs were observed in 4 (40%) and 1 (33%) patients at the 2 and 4 mg/kg dose levels, respectively. At the 2.0 mg/kg dose level, serious TEAEs were gingival infection; pneumonia, hyponatremia; cholangitis, hyperbilirubinemia; and proteinuria (1 event each). At the 4.0 mg/kg dose level, serious TEAEs were tumor pain, decreased appetite, reversible posterior leukoencephalopathy syndrome, and nausea (1 event each). There were no serious TEAEs that occurred in more than 1 patient. Grade 4 hypertension was not reported. Grade 3/4 hematological abnormalities of leukocytes, neutrophils, activated partial thromboplastin time, and lymphopenia were observed in 1 patient each (10%) at the 2.0 mg/kg dose level, and no grade 3/4 abnormality was observed at the 4.0 mg/kg dose level. Regarding grade 3/4 biochemical abnormalities, bilirubin (10%), aspartate aminotransferase (20%), alanine aminotransferase (10%), hypertriglyceridemia (10%), hyponatremia (10%), and hyperglycemia (10%) were observed at the 2.0 mg/kg dose level, and bilirubin (33%) was observed at the 4.0 mg/kg dose level. There were no treatment-related deaths.

**Table 2:** Incidence of the most common treatment emergent adverse events^a,b^

Preferred term,^c^ n (%)	Aflibercept dose level			
	2 mg/kg (N = 10)		4 mg/kg (N = 3)	
	All grades	Grade 3/4	All grades	Grade 3/4
Decreased appetite	10 (100)	1 (10)	2 (67)	1 (33)
Hypertension	7 (70)	4 (40)	3 (100)	2 (67)
Diarrhea	8 (80)	0	1 (33)	0
Fatigue	7 (70)	2 (20)	1 (33)	1 (33)
Nausea	7 (70)	0	1 (33)	0
Constipation	6 (60)	0	1 (33)	0
Stomatitis	6 (60)	0	0	0
Weight decreased	5 (50)	0	1 (33)	0
Epistaxis	5 (50)	0	1 (33)	0
Proteinuria	4 (40)	1 (10)	2 (67)	1 (33)

^a^Reported in ≥6 patients overall at any grade

^b^One patient can have more than 1 adverse event

^c^Adverse events are reported according to the Medical Dictionary for Regulatory Activities version 13.1 and graded using National Cancer Institute Common Toxicity Criteria version 3.0

### Pharmacokinetics and immunogenicity

**Fig. 1:**
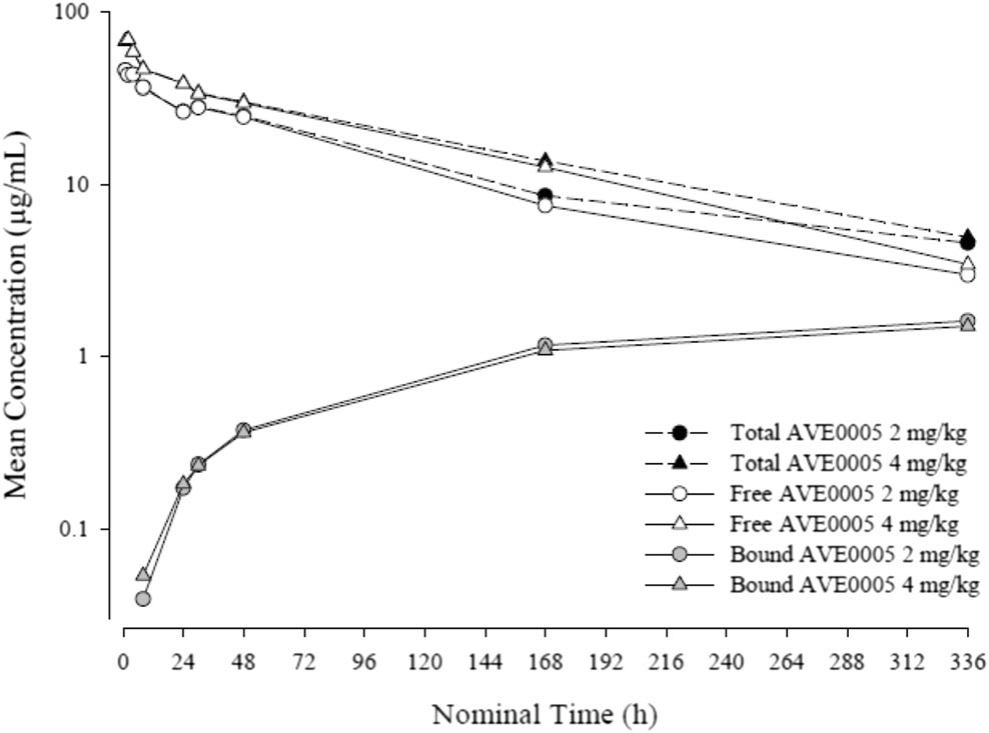
Mean plasma concentration versus time profiles of free, adjusted-bound and total aflibercept on day 1 of cycle 1 following a single administration (semi-log scale)

**Fig. 2:**
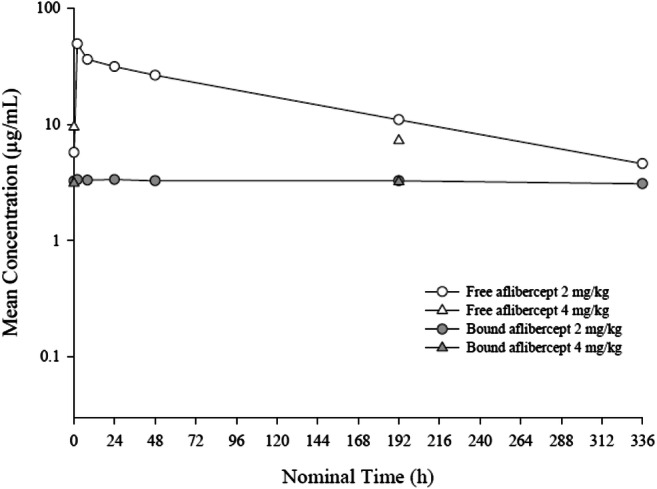
Mean plasma concentration versus time profiles of free and adjusted-bound aflibercept on day 42 of cycle 1 following multiple dosing in the presence of S-1 (semi-log scale)

PK parameters of free and VEGF-bound aflibercept in cycle 1 after a single administration at 2 mg/kg or 4 mg/kg are summarized in Table 3. PK parameters of free and VEGF-bound aflibercept at 2 mg/kg or 4 mg/kg in the presence of S-1 in cycle 1 on day 42 after repeated administrations are summarized in Table 4. In the presence of S-1 (day 42), free aflibercept had a mean half-life of 5.05 and 4.28 days at 2 and 4 mg/kg, respectively, and the median maximum free aflibercept concentration was observed approximately 3 h post-dosing (2 h after the end of the infusion). In the absence of S-1 (day 1), free aflibercept had a mean half-life of 3.77 and 3.86 days at doses of 2 and 4 mg/kg, respectively, and the median maximum free aflibercept concentration was observed approximately 2 h post-dose (1 h after the end of the first infusion). At the dose level of 2 mg/kg, free aflibercept was eliminated with a mean clearance of 0.759 L/day. In the presence of S-1, the mean clearance at steady state was similar, at 0.649 L/day. At the dose level of 4 mg/kg, free aflibercept was eliminated with similar clearances in the presence and absence of S-1. The volume of distribution at steady state was approximately 4 L across aflibercept dose levels regardless of the presence or absence of S-1. Mean free and VEGF-bound plasma concentrations on day 1 of cycle 1 for each dose level cohort are illustrated in Fig. 1. Mean plasma concentration versus time profiles of free, adjusted-bound and total aflibercept on day 42 in cycle 1 after multiple dosing in the presence of S-1 are presented in Fig. 2. The mean (± standard deviation) PK parameters of S-1 analytes (5-FU, CDHP, tegafur and oteracil) on day 42 after repeated twice daily oral administration of S-1 for two weeks are summarized in Table 5.

**Table 3:** Plasma pharmacokinetic parameters of free and VEGF-bound aflibercept in cycle 1 following single administration at 2 mg/kg or 4 mg/kg

	Free aflibercept		VEGF-bound aflibercept	
	Aflibercept dose level			
Mean ± SD (Geometric mean) [CV%]	2 mg/kg	4 mg/kg	2 mg/kg	4 mg/kg
Number of patients	10	3	10	3
C_max_, μg/mL	52.5 ± 24.6 (48.0) [46.8]	70.2 ± 5.94 (70.0) [8.5]	1.60 ± 0.717 (1.48) [44.7]	1.50 ± 0.298 (1.48) [19.9]
T_max_^a^, days	0.08 (0.04–0.17)	0.08 (0.04–0.08)	13.97 (13.79–14.02)	13.98 (7.00–16.10)
AUC_last_, μg**·**day/mL	173 ± 56.2 (166) [32.4]	247 ± 51.7 (243) [21.0]	13.4 ± 5.44 (12.5) [40.6]	13.8 ± 1.16 (13.7) [8.5]
AUC_0–336_, μg**·**day/mL	153 ± 32.2 (150) [21.0]^b^	243 ± 46.6 (240) [19.2]	12.3 ± 6.71 (11.4) [54.4]^c^	NC ± NC (NC) [NC]
AUC, μg**·**day/mL	166 ± 35.0 (163) [21.1]^b^	269 ± 67.7 (263) [25.2]	NC ± NC (NC) [NC]	NC ± NC (NC) [NC]
t_1/2z_, day	3.77 ± 0.858 (3.68) [22.7]^b^	3.86 ± 1.47 (3.63) [38.1]	NC ± NC (NC) [NC]	NC ± NC (NC) [NC]
CL, L/day	0.759 ± 0.253 (0.730) [33.3]^b^	0.917 ± 0.196 (0.903) [21.3]	NA	NA
V_ss_, L	3.53 ± 0.835 (3.43) [23.7]^b^	4.57 ± 1.13 (4.48) [24.7]	NA	NA

*CV%*, coefficient of variation percentage; *NA*, not applicable; *NC*, not calculated; *SD*, standard deviation; *VEGF*, vascular endothelial growth factor. PK parameters reported are: *AUC*, area under the concentration versus time curve extrapolated to infinity; *AUC*_*0–336*_, AUC from time 0 to 336 h; *AUC*_*last*_, AUC from time 0 to the real time t_last_; *CL*, total body clearance; *C*_*max*_, maximum drug concentration observed; *t*_*1/2z*_, terminal half-life; *T*_*max*_, first time to reach C_max_; *V*_*ss*_, apparent volume of distribution at steady state

^a^Median (range)

^b^N = 8 (two patients were not evaluable)

^c^N = 2 (eight patient were not evaluable)

**Table 4:** Plasma pharmacokinetic parameters of free and VEGF-bound aflibercept on day 42 following repeated administrations at 2 mg/kg or 4 mg/kg in the presence of S-1

	Free aflibercept		VEGF-bound aflibercept	
	Dose level			
Mean ± SD (Geometric mean) [CV%]	2 mg/kg	4 mg/kg^a^	2 mg/kg	4 mg/kg^a^
Number of patients	8	1	8	1
C_max_, μg/mL	49.6 ± 18.7 (47.0) [37.7]	77.5	3.72 ± 1.36 (3.49) [36.6]	2.91
T_max_^b^, days	0.13 (0.13–0.37)	0.13	1.04 (0.37–8.93)	9
AUC_last_, μg**·**day/mL	242 ± 134 (218) [55.3]	250	40.6 ± 12.1 (39.1) [29.7]	44
AUC_τ_, μg**·**day/mL	232 ± 125 (211) [53.9]	242	39.3 ± 12.1 (37.9) [30.9]^c^	37.9
t_1/2z_, day	5.05 ± 1.66 (4.78) [32.9]	4.28	NC ± NC (NC) [NC]^d^	NC
CL_ss_, L/day	0.649 ± 0.397 (0.579) [61.2]	0.813	NA	NA
V_ss_, L	3.83 ± 0.987 (3.71) [25.7]	4.19	NA	NA

*CV%*, coefficient of variation percentage; *NA*, not applicable; *NC*, not calculated; *SD*, standard deviation; *VEGF*, vascular endothelial growth factor. PK parameters reported are: *AUC*_*last*_ area under the concentration versus time curve (AUC) from time 0 to the real time t_last_; *AUC*_*τ*_, AUC from time 0 to the end of the dosing period; *CL*_*ss*_, total body clearance at steady state; *C*_*max*_, maximum drug concentration observed; *t*_*1/2z*_, terminal half-life; *T*_*max*_, first time to reach C_max_; *V*_*ss*_, apparent volume of distribution at steady state

^a^N = 1, individual values listed

^b^Median (range)

^c^N = 5 (three patients were not evaluable)

^d^N = 0 (none of the patients were evaluable)

**Table 5:** Mean pharmacokinetic parameters of S-1 analytes on day 42 following repeated twice daily oral administration of S-1 for two weeks

S-1 analyte								
	5-FU		CDHP		Tegafur		Oteracil	
	Aflibercept dose level							
Mean ± SD	2 mg/kg	4 mg/kg^a^	2 mg/kg	4 mg/kg^a^	2 mg/kg	4 mg/kg^a^	2 mg/kg	4 mg/kg^a^
Number of patients	7	1	7	1	7	1	7	1
C_max_, ng/mL	130 ± 35.0	104	367 ± 138	266	4730 ± 1840	4670	53.5 ± 38.7	29.5
T_max_^b^, h	2.00 (1.97–2.05)	3.95	1.05 (0.97–2.05)	1.97	1.05 (0.92–2.05)	1.97	1.97 (0.97–2.05)	3.95
AUC_τ_, ng**·**h/mL	912 ± 174^c^	654	1650 ± 540^e^	1320	41,600 ± 19,500	47,100	306 ± 269^g^	226
AUC_last_, ng**·**h/mL	788^c^ ± 370	817	1820^e^ ± 825	1730	91,400 ± 52,600	113,000	378^g^ ± 390	466
t_1/2z_, h	3.66 ± 0.0225^d^	3.87	3.63 ± 1.13^f^	9.2	15.7 ± 4.26	19.6	5.72 ± 3.48^h^	19.6

*CDHP*, gimeracil; *5-FU*, 5-fluorouracil; *SD*, standard deviation. PK parameters reported are: *AUC*_*last*_ area under the concentration versus time curve (AUC) from time 0 to the real time t_last_; *AUC*_*τ*_, AUC from time 0 to the end of the dosing period; *C*_*max*_, maximum drug concentration observed; *t*_*1/2z*_, terminal half-life; *T*_*max*_, first time to reach C_max_

^a^n = 1, individual values listed

^b^Median (range)

^c^*n* = 3, 4 patients were not included in the calculation of summary statistics

^d^n = 2, 5 patients were not included in the calculation of summary statistics

^e^n = 6, 1 patient was not included in the calculation of summary statistics

^f^n = 4, 3 patients were not included in the calculation of summary statistics

^g^n = 6, 1 patient was not included in the calculation of summary statistics

^h^n = 4, 3 patients were not included in the calculation of summary statistics

Profile of 1 patient was excluded for 5-FU, CDHP, tegafur and oteracil

All 13 patients treated with aflibercept were evaluable for immunogenicity. Two out of 10 (20%) patients at the 2 mg/kg dose level and 1 out of 3 (33%) patients at the 4 mg/kg dose level were found to be positive for aflibercept antibodies both at baseline and post-baseline.

### Antitumor activity

Eight of 13 patients (62%) had a best overall response of stable disease (SD), including 6 of 10 (60%) at the 2 mg/kg dose level and 2 of 3 (67%) at the 4 mg/kg level. The primary tumor sites for these 8 patients were rectum (*n* = 6), colon (*n* = 1) and stomach (n = 1). There were no patients with a complete response (CR) or partial response (PR). The median number of cycles with aflibercept administration per patient was 3.0 (range, 1 to 11) at the 2.0 mg/kg dose level and 1.0 (range, 1 to 2) at the 4.0 mg/kg dose level, with an aflibercept median relative dose intensity (RDI) of 0.903 (range, 0.71 to 1.01) at the 2.0 mg/kg dose level and 0.748 (range, 0.25 to 0.79) at the 4.0 mg/kg dose level. Median exposure to S-1 at aflibercept 2.0 mg/kg and 4.0 mg/kg dose levels were 3.0 (range, 1 to 11) and 1.5 (range, 1 to 2) cycles per patient with a median RDI of 0.771 (range, 0.60 to 0.95) and 0.763 (range, 0.73 to 0.80), respectively.

## Discussion

The objective of the current study was to define a dose of aflibercept that could be safely administered with S-1 in Japanese patients with advanced solid malignancies for further investigation. The initial plan was that for sequential cohorts of 3–6 patients, the dose level of aflibercept would be escalated from a starting level of 2 mg/kg, while the dose level of S-1 was held constant at 40 mg/m^2^ twice daily. However, because one of the first 3 patients treated at the second dose level of 4 mg/kg developed a DLT and another patient developed RPLS outside the DLT assessment period, we decided to reassess tolerability and safety at the 2.0 mg/kg dose level before proceeding to the 4.0 mg/kg dose level. Subsequently, this study was terminated by the sponsor in consideration of global development status of S-1 treatment for gastric cancer and the progress of this study. The MTD was therefore not reached in this study and a RP2D for aflibercept in combination S-1 in Japanese patients was not defined. However, based on the available data from patients enrolled at the initial dose level, we concluded that aflibercept 2 mg/kg given every 2 weeks in combination with S-1 was tolerable in Japanese patients.

All 13 patients who were assessed for safety experienced at least 1 TEAE. Most of these TEAEs were manageable and predominantly of low grade except for grade 3 hypertension observed at in 6 (46%) of all 13 patients. Hypertension and proteinuria (seen at grade 3/4 in 2 [15%] of 13 patients) are commonly associated with anti-VEGF agents [20]. There were no treatment-related deaths. Serious TEAEs were observed in 4 (40%) and 1 (33%) patients at the 2 and 4 mg/kg dose levels, respectively.

A positive value in the anti-drug antibody (ADA) assay was detected in 3 patients (2 patients at the 2 mg/kg dose level and 1 patient at the 4 mg/kg dose level); these 3 patients also had a positive value for ADA at baseline. Although the reasons why patients were positive for ADA before aflibercept was administered are not obvious, this should be investigated before the use of aflibercept in Japanese patients is considered. However, our data suggest that aflibercept might not be highly immunogenic in Japanese patients because ADA did not appear after the administration of aflibercept. No major allergic reactions, such as anaphylactic shock, bronchospasm, generalized urticaria, or infusion related reactions were observed in this study.

Plasma concentrations of free aflibercept and its associated PK parameters were unaffected by repeated dosing of aflibercept in the presence of S-1 at the 2 mg/kg dose level. PK parameters were comparable with those previously reported in a Western population with advanced solid tumors or non-Hodgkin’s lymphomas receiving treatment with aflibercept [21]. The limited data at an aflibercept dose of 4 mg/kg, followed the same trends as those for 2 mg/kg, however, obtained data was insufficient to draw firm conclusions on dose response.

In terms of efficacy, 6 patients at the 2 mg/kg dose level and 2 patients at the 4 mg/kg dose level had SD as a best overall response. There were no CRs or PRs at either dose level in this heavily pretreated population. The limited efficacy observed in this study may be attributed to the low aflibercept dose administered to most patients.

In conclusion, though the MTD was not reached for aflibercept in combination with S-1 in Japanese patients since the study was terminated prematurely, the tolerability and safety of aflibercept at the 2 mg/kg dose level in combination with S-1 was shown, based on DLT incidence and the overall safety profile.
